# Proteomic analysis of a *Candida albicans pir32* null strain reveals proteins involved in adhesion, filamentation and virulence

**DOI:** 10.1371/journal.pone.0194403

**Published:** 2018-03-19

**Authors:** Pamela El Khoury, Andy Awad, Brigitte Wex, Roy A. Khalaf

**Affiliations:** Department of Natural Sciences Lebanese American University, Byblos, Lebanon; Yonsei University, REPUBLIC OF KOREA

## Abstract

We have previously characterized Pir32, a *Candia albicans* cell wall protein that we found to be involved in filamentation, virulence, chitin deposition, and resistance to oxidative stress. Other than defining the cell shape, the cell wall is critical for the interaction with the surrounding environment and the point of contact and interaction with the host surface. In this study, we applied tandem mass spectrometry combined with bioinformatics to investigate cell wall proteome changes in a *pir32* null strain. A total of 16 and 25 proteins were identified exclusively in the null mutant strains grown under non-filamentous and filamentous conditions. These proteins included members of the PGA family with various functions, lipase and the protease involved in virulence, superoxide dismutases required for resisting oxidative stress, alongside proteins required for cell wall remodeling and synthesis such as Ssr1, Xog1, Dfg5 and Dcw1. In addition proteins needed for filamentation like Cdc42, Ssu81 and Ucf1, and other virulence proteins such as Als3, Rbt5, and Csa2 were also detected. The detection of these proteins in the mutant and their lack of detection in the wild type can explain the differential phenotypes previously observed.

## 1. Introduction

*Candida albicans* is normally found in many homeotherms as a benign commensal organism residing asymptomatically on mucosal surfaces and on the skin [1]. However, following an ecological shift or disturbance in the microbial flora, *C*. *albicans* becomes an opportunistic pathogen. Factors causing such disturbance include the uptake of broad-spectrum antibiotics, hormonal imbalances, malnutrition, and excessive carbohydrates intake [2]. Patients of radiotherapy, chemotherapy, xerostomia, organ transplantation, endocrine disorders, or HIV infection are at risk of developing a *C*. *albicans* infection ranging from local to systemic candidiasis [1, 3]. Statistically, *C*. *albicans* is among the most frequently identified agents causing nosocomial infections and the third most frequently isolated pathogen from the bloodstream as per the rankings of the Center for Disease Control [4]. *C*. *albicans* has been reported to cause 250 to 400 thousand deaths per year worldwide [5]. Several factors contribute to the transition of *C*. *albicans* between commensalism and pathogenicity.

This transition is governed in large parts by the ability of *C*. *albicans* to interchange between 2 morphologies: yeast and hyphal forms. Both forms are crucial for the development and maintainance of *C*. *albicans* pathogenicity (Jacobsen et al., 2012). The first form is required for the attachment to host cells and subsequent dissemination and the latter form is required for tissue invasion and biofilm formation [6]. Yeast-to-hyphae switching is triggered by various environmental conditions including hypoxia, temperature of 37°C, serum availability, neutral to basic pH, glucose depletion, and contact with a host cell [7]. In both morphologies, the cells are bound by a cell wall that is continuously modified upon morphogenesis and growth. The cell wall is formed of an inner chitin layer, a β-1,3-glucan layer, a β-1,6-glucan layer, and an outer mannan layer [8]. The fungal cell wall comprises 30% of the overall cell dry weight, where polysaccharides correspond to almost 90% of which, and the remainder is represented by proteins [9]. These proteins have various functions. Proteins attached to the outer surface are related to host cell adhesion such as lectins and adhesins. Some cell wall proteins, like lipases and proteases, degrade the external structures of the host cells and tissues thus aiding in invasion. Others, such as chitinases and mannosidases, are engaged in the synthesis and remodeling of the cell wall itself thus affecting its rigidity and resistance to stresses. Additionally, some proteins are needed to escape the host defenses such as superoxide dismutases [10].

One group of wall proteins, called the Pir proteins standing for proteins with internal repeats, are attached to the β-1,3-glucan layer by alkali-labile bonds and are highly O-glycosylated. These proteins lack a glycosyl phosphatidyl inositol (GPI) anchor motif, but include a signal peptide, internal repeats, a sensitive site for Kex2, and 4 Cys residues at the C-terminal sequence [11]. In *C*. *albicans*, there are two Pir proteins: Pir1 and Pir32. Pir1 was found to be an essential protein, required for the stability and rigidity of the cell wall [12].

Pir32 is a 422 amino acid long protein previously characterized in our lab by homologous recombination, a *pir32* null strain [13]. Pir32 was found to be involved in virulence, chitin deposition, stress response, and filamentation. Since this protein is located at the cell surface, we hypothesized that the lack of Pir32 would be reflected in various defects in the cell wall proteome and associated pathways. When compared to the wild type strain, the *pir32* null strain exhibited hyperfilamentation, enhanced virulence, doubled chitin content, and elevated response to stresses [13]. Accordingly, the aim of this study was to analyze the cell wall proteome of the *pir32* null strain in order to explain the observed mutant phenotypes. As such, cell walls from the wild type and mutant strains, under filamentous and non-filamentous growth conditions, were isolated and various chemicals and enzymes were added to fractionate the proteins on the basis of their cell wall anchorage. Tandem-MS, following tryptic digestion of the isolated proteins, was performed followed by data analysis in order to identify the cell wall proteins that are exclusive to each strain. A similar approach was applied in our lab to further characterize another *C*. *albicans* cell wall protein, Dse1 [14]. Additionally, similar methods have been previously developed to study the way *C*. *albicans* cell wall is influenced by surface stress, and to investigate the plasma membrane proteome and secretome [15–17].

## 2. Materials and methods

### 2.1 Strains utilized

The *C*. *albicans* wild type strain RM1000 (*ura3*Δ::_*imm434/ura3*Δ::_*imm434his1*::*hisG/his1*::*hisG*) which is a histidine and uridine auxotroph was transformed with plasmid pABSK2 which contains a functional *URA3* gene, and the null strain *pir32*:*URA3/pir32*::*HIS1*, were utilized in this study [13].

### 2.2 Media preparation and culture conditions

The above mentioned strains were grown at 28°C on potato dextrose agar plates (Oxoid) containing potato extract, glucose, and agar and supplemented with histidine and uridine. Then, few colonies were collected from each plate and were grown in a liquid media of rich potato dextrose broth (PDB) (Hi Media, India) until exponential phase. For non-filamentous growth, strains were incubated overnight at 28°C under aerobic condition. For filamentous growth, PDB media was supplemented with 10% fetal bovine serum (Gibco) and incubated overnight at 37°C under aerobic condition.

### 2.3 Cell wall isolation and protein extraction

Twelve cell wall extractions from each strain and for growth condition were performed independently. Cells were first centrifuged at 4,000 rpm for 5 min, then re-suspended in 5 mL Tris (5 mM, pH = 7.8). Protease Inhibitor Cocktail (6 μL, abcam ab65621) along with cold glass beads were added in a 1:1 beads to pellet volume ratio. Thirty cycles of vortexing were applied to ensure breakage as follows: 30 sec on vortex followed by 30 sec on ice. Samples turned orange reflecting a reaction between the acidic cytosol and the Protease inhibitor. Beads were then removed by pouring the samples into other pre-weighed tubes and washing the beads several times with cold NaCl until the supernatant becomes transparent; making sure not to transfer any beads to the new pre-weighed tubes during the process. The efficiency of breakage was examined under the microscope. The samples were spun at 3,000 rpm for 5 min, **t**he supernatants containing intracellular proteins were poured off, while pellets were re-suspended in NaCl (40 mL, 1 M) and spun again at 3,000 rpm for 5 min. This NaCl washing step was repeated three to four times. Protein extraction buffer (50 mM Tris, 2% SDS, 100 mM Na-EDTA, 150 mM NaCl, pH 7.8) with β-mercaptoethanol (8 μL per 1 mL protein extraction buffer) was added (0.5 mL buffer per 100 mg wet weight walls) and the pellets were re-suspended. Tubes were boiled for 10 min and spun for 5 min at 3,000 rpm. The supernatants containing the SDS extractable proteins were collected and later subjected to tryptic digestion and subsequent steps for protein identification. Protein extraction buffer and β-mercaptoethanol were added again as before to re-suspend pellet. Samples were boiled, cooled, centrifuged for 5 min at 3,000 rpm, and re-suspended in water. Wash steps with Type 1 water were done to remove excess SDS. The final pellets were frozen in liquid N_2_ and freeze-dried. Lyophilized cell walls were finally stored at -20°C until use.

### 2.4 Extraction of alkali labile cell wall proteins

The cell wall pellets were subjected to overnight incubation with NaOH (30 mM) at 4°C. They were then neutralized with aqueous acetic acid (30 mM) [18]. Samples were spun at 3,000 rpm for 5 min, and supernatants were collected and subjected to tryptic digestion.

### 2.5 Glucanase treatment of cell wall pellets

Spectrophotometric analysis was used to estimate cell numbers. 10^8^ cells were incubated at 37°C with 1 mg of Glucanase (β-(1–3)-D-Glucanase from *Helix pomatia*, Sigma-Aldrich) in sodium acetate buffer (1 mL, 150 mM, pH 5) overnight [19]. Supernatants were collected and subjected to tryptic digestion.

### 2.6 Tryptic digestion

All cell wall protein extracts were incubated in a reducing buffer (10 mM DTT, 100 mM NH_4_HCO_3_) at 55°C for 1 h. Samples were cooled to room temperature and spun at 3,000 rpm for 5 min. An alkylating buffer (65 mM iodoacetamide, 100mM NH_4_HCO_3_) was added to the pellets that were kept for 45 min at room temperature in the dark. Subsequently, a quenching solution (55 mM DTT, 100 mM NH_4_HCO_3_) was added to the samples for 5 min at room temperature. Ammonium bicarbonate buffer (50 mM) was used to wash the samples 5 times. Pellets were re-suspended in solution containing ammonium bicarbonate (50 mM) and trypsin (1μg/μL; Sigma-Aldrich). Samples were left at 37°C for 16 h. Then, they were spun at 3,000 rpm for 5 min, and the supernatants were collected and prepared for Zip Tipping by adding TFA (0.1% V/V).

### 2.7 Peptide concentration

ZipTip C18 clean up tips (Millipore^®^ Ziptips, Sigma-Aldrich, 0.6 μL C18 resin, volume 10 μL) were wetted in acetonitrile solution and then equilibrated in a 0.1% TFA HPLC water solution. Sample binding was achieved by full pressing the pipette a minimum of 10 times in the digest tube. The membrane was then washed in a 0.1% TFA HPLC water solution. Sample elution was performed using 10 μL of elution buffer (0.1% TFA (v/v) in HPLC water/acetonitrile (1:1).

### 2.8 MALDI TOF/TOF

Digested cell wall proteins were spotted on a stainless steel target plate (Opti-TOF TM 384 Well Insert, 128x81 mm RevA, Applied Biosystems). BSA digests were also spotted and used as a standard solution. The sample and BSA digest spots were overlaid with α-cyano-4-hydroxy-cinnamic acid matrix solution (10 mg CHCA matrix in 50% acetonitrile with 0.1% TFA) and air-dried. MALDI-TOF-TOF MS spectra were acquired on 4800 MALDI-TOF-TOF analyzer (operated by the 4000 Series Explorer software version 3.7). The instrument was externally calibrated using TOF/TOF Calibration Mixture (Mass Standards Kit for Calibration of AB SCIEX TOF/TOF^™^ Instruments). MS reflector positive mode at a laser intensity of 2500 was used as an acquisition method. The selected mass range was 499 Da-2500 Da with a focus mass of 1500 Da. Reflector positive default was used as a processing method with a minimum signal-to-noise ratio of 5. The resulting mass lists were manually scanned for known contaminant mass peaks including keratin, matrix, and trypsin autolysis. The identified contaminant mass peaks were used to create an exclusion list that applied in the interpretation method for the MS/MS data acquisition. The minimum signal-to-noise filter for the monoisotopic precursor selection for MS/MS was also assigned 5. “Strongest precursors first” option was selected for precursor sorting order per spot and “weakest precursors first” option was selected for MS/MS acquisition order per spot with a maximum of 30 precursors per spot for each. MS/MS 1kV positive was used as an MS/MS acquisition method with a fixed laser intensity of 3500 and a precursor mass of 1570.677 Da. CID was turned on with specifying medium gas pressure and air gas type. Metastable suppressor was also turned on. MS/MS positive default was used as an MS/MS processing method with a signal-to-noise threshold of 5 for monoisotopic peaks.

### 2.9 Protein identification

MS/MS Ion Search was performed first using the contaminants and cRAP databases, in order to eliminate any additional contaminants whose peaks were not included within the exclusion list, then using a custom database on the MASCOT Server in order to identify the proteins within the samples. The database consisted of protein sequences of all curated *C*. *albicans* proteins (taxon id: 237561) present in the Swissprot database (2016_07, retrieved on September 18, 2016) with gene ontology: cell wall (GO: 0005618), plasma membrane (GO: 0005886), and transmembrane (GO: 0016021) localization tags. The peptide and fragment tolerance values were specified at ±2 Da. This may seem too tolerant; however, the default settings of the 4800 MALDI-TOF-TOF analyzer were used where the resolution per mass peak as displayed by the machine is on average 4000 which is lower than the preferred acceptable values. This is another limitation of the machine. As such we had to choose a slightly more lenient tolerance level. Carbamidomethyl C was chosen as a fixed modification, whereas Oxidation at M was selected as a variable modification. Up to two missed cleavages were permitted for trypsin. A peptide charge of 1+ was assigned and MALDI-TOF-TOF was picked in the instrument type option. After these parameters were assigned, the data file was chosen and searched.

In the exported peptide summary report generated by MASCOT, proteins with less than 2% sequence coverage were disregarded. The nature of the work MS/MS Ion Search results in greater peptide confidence than PMF, which makes the issue of sequence coverage less important. 2% coverage equates to an average of 12 amino acids identified in a specific order which is enough for unmistakable protein identification (Barrett et. al, 2005). We added the 2% cut off as an extra step to reduce false positives.

Peptide sequences identified by MASCOT but not assigned to any protein were blasted on Candidagenome.org where the selected target genome and target sequence dataset were “*Candida albicans* SC5314 Assembly 22” and “Proteins—translation of coding sequence (PROTEIN)” respectively. The BLASTP program was selected and no gapped alignments were allowed. The cut off e-value was < 0.05 for MASCOT searches as well as BLAST searches.

## 3 Results

**Fig 1:**
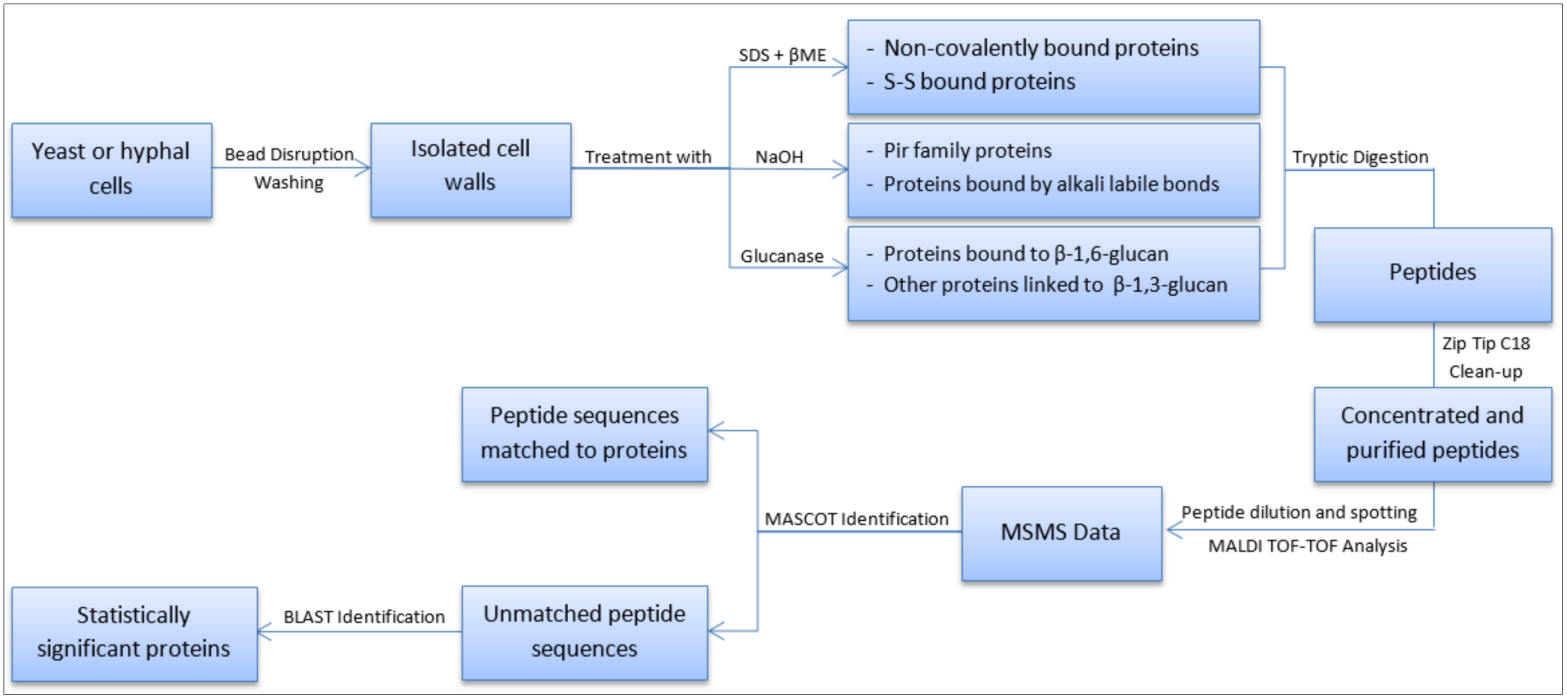
Graphical representation of the experimental procedure followed in this study in order to extract the cell wall proteins and subsequently identify them in the wild type and the *pir32* null strains.

Cell wall proteins were extracted from *C*. *albicans* strains that were grown under either filamentous or non-filamentous conditions as illustrated in Fig 1. These proteins were identified by analyzing the MS/MS data acquired by MALDI TOF/TOF using MASCOT. When peptide sequences were not assigned to any protein, the obtained sequences were identified by blasting on the *Candida* genome database (candidagenome.org).

Our customized database contains a total of 218 verified proteins. Prior to any comparison, we were able to identify under nonfilamentous growth conditions 123 proteins in the wild type strain and 92 in the *pir32* null strain, and under filamentous growth conditions 119 proteins in the wild type strain and 152 in the *pir32* null strain. This shows that we have successfully identified almost 52% of the predicted cell wall proteins.

In total 56 proteins, listed in Table 1, were common between the wild type and the null mutant strains under all growth conditions; 10 of which are known to be essential, and thus expected to be detected under all growth conditions. In this study, the identified proteins that were only detected in the *pir32* null strain are divided into two categories depending on the method utilized to identify them. Proteins identified through MASCOT are listed in Tables 2 and 3, and proteins identified through BLAST are listed in Tables 4 and 5. Additionally, proteins exclusively detected in the wild type strain are presented in Table 6, and those that are hyphae-specific and differentially detected in the studied stains between nonfilamentous growth and filamentous growth are listed in Tables 7 and 8.

**Table 1:** Common proteins between the wild type and the null mutant strains regardless of the growth conditions.

GENE NAME	description
***AIM36***	Altered inheritance of mitochondria protein 36
***ALO1***	D-Arabinono-1,4-lactone oxidase
***APE2***^a^	Neutral arginine, alanine, leucine specific metallo-aminopeptidase
***BMH1***	14-3-3 protein homolog
***BNA4***	Putative kynurenine 3-monooxygenase
***BUD2***	GTPase activating protein for Rsr1
***BUD4***	Bud site selection protein B
***CDC11***	Septin
***CDC19***	Pyruvate kinase at yeast cell surface
***CDR1***	Multidrug transporter of ABC superfamily
***EFT2***^a^	Elongation Factor 2
***END3***	Actin cytoskeleton-regulatory complex protein
***ENG1***	Endo-1,3-beta-glucanase
***ENO1***^a^	Enolase
***ERG11***	Lanosterol 14-alpha-demethylase
***ERG6***	Delta(24)-sterol C-methyltransferase
***GPM1***	Phosphoglycerate mutase
***HSP21***	Small heat shock protein
***HSP90***^a^	Heat shock protein 90
***HWP2***	GPI-anchored, glycosylated cell wall protein
***INO1***	Inositol-1-phosphate synthase
***IPP1***^a^	Putative inorganic pyrophosphatase
***LIP10***	Secreted lipase
***LIP8***	Secreted lipase
***MDR1***	Plasma membrane MDR/MFS multidrug efflux pump
***MET6***^a^	5-methyltetrahydropteroyltriglutamate-homocysteine methyltransferase
***MIC60***	MICOS complex subunit
***MLT1***	Vacuolar membrane transporter
***MNT1***	Alpha-1,2-mannosyl transferase
***PAN1***^a^	Actin cytoskeleton-regulatory complex protein
***PDC11***	Pyruvate decarboxylase
***PGA41***	Putative GPI-anchored adhesin-like protein
***PGK1***	Phosphoglycerate kinase
***PHR1***	pH-responsive protein 1, Glycosidase
***PHR2***	pH-responsive protein 2, Glycosidase
***PMT2***^a^	Protein mannosyltransferase
***PRM1***	Putative membrane protein
***PRT1***	Putative translation initiation factor eIF3
***SAP4***	Secreted aspartyl proteinase
***SAP6***	Biofilm-specific aspartyl protease
***SAP7***	Pepstatin A-insensitive secreted aspartyl protease
***SAP8***	Secreted aspartyl protease
***SAP9***	Secreted aspartyl protease
***SEC4***	Small GTPase of Rab family
***SEC62***	Putative endoplasmic reticulum protein-translocation complex subunit
***SLN1***	Histidine protein kinase
***SSA1***	HSP70 family chaperone
***SSA2***	HSP70 family chaperone
***SSC1***^a^	Heat shock protein
***SSU1***	Sulfite transport protein
***STP3***	sulfite transport protein
***TEF1***	Transcription factor
***TIM21***	Component of the translocase of the inner mitochondrial membrane
***TIM50***	Component of the translocase of the inner mitochondrial membrane
***UGP1***	UTP-glucose-1-phosphaturidyl transferase
***VMA2***	Vacuolar H(+)-ATPase

^a^ Essential protein

**Table 2:** Proteins, extracted from cells of the *pir32* null strain grown under nonfilamentous conditions, identified through MASCOT.

Gene Name	Protein accession	Protein description	Sequence coverage (%)	Peptide sequence(s)	# Missed cleavages
***DFG5***	Q5ACZ2	Mannan endo-1,6-alpha-mannosidase	3.8	NSISNGCLFHLAARLAR	1
***DSE1***	Q59Y20	Protein DSE1	3.5	LSANIVK	0
				KPTTTIK	0
				DSQWEENDNTK	0
***LIP7***	Q9P4E7	Lipase 7	3.3	DLETSQLGDVLKWR	1
***NCE102***	Q5ANE3	Non-classical export protein 102	7.6	GGLFGHSSRPAPR	0
***PGA27*** ^a^	Q5AD23	Predicted GPI-anchored protein 27	2.9	TDIPVSQRIDTISK	1
***SAP2***	P0DJ06	Candidapepsin-2	3.3	SAGFVALDFSVVK	0

^a^ Protein identified through BLAST search as well from cells grown under same conditions.

**Table 3:** Proteins, extracted from cells of the *pir32* null strain grown under filamentous conditions, identified through MASCOT.

Gene Name	Protein accession	Protein description	Sequence coverage (%)	Peptide sequence	# Missed cleavages
***ALS3***	Q59L12	Agglutinin-like protein 3	3.7	AGTNTVTFNDGDKK	1
				CFKAGTNTVTFNDGDK	1
				ISINVDFER	0
				NSDAGSNGIVIVATTR	0
***CSA2***	Q5A0X8	Surface antigen protein 2	7.5	CRGADVTNFRK	2
***DFI1***^a^	Q5AFI4	Cell-surface associated glycoprotein	8.3	LGSDEFFNGELGVR	0
				RNNRDYEGGWTFWR	2
***ERV25***	Q5A302	Endoplasmic reticulum vesicle protein 25	9.3	AREERMR	2
				VTDSLGNEYRNKK	2
***LIP9***	Q9P4E6	Lipase 9	2.6	NATVGDILQFRK	1
***PGA17***	Q5AHA4	Predicted GPI-anchored protein 17	2.7	NIVGEVVIEALPLIK	0
***PGA31***	Q5A5U9	Cell wall protein	2.4	DSYAVVK	0
***PGA49*** ^a^	Q59QA5	Predicted GPI-anchored protein 49	4.7	QPVSLSMPTK	0
				NRSKFYNHFLR	2
				LHSPTSTDTKSSK	1
***RBT5*** ^a^	Q59UT4	Repressed by TUP1 protein 5	9.1	IYDQLPECAK	0
				AEETSKAAETTK	1
***RPL13*** ^a^	O59931	60S ribosomal protein L13	11.9	TIGISVDHRRQNK	2
				QNKSQETFDANVAR	1
***SSR1***	Q5AFN8	Covalently-linked cell wall protein 14	3.8	NSDVEKCLK	1
***STP2***	Q5AL16	Transcriptional regulator STP2	2.1	MIDPELVPFASK	0

^a^ Protein identified through BLAST search as well from cells grown under same conditions.

**Table 4:** Proteins, extracted from cells of the *pir32* null strain grown under nonfilamentous conditions, identified through BLAST.

Gene Name	Description	Sequence	E-value	% Match
**C1_00310W_A**	Putative protein of unknown function	DPEIFQMTNR	2.00E-05	100%
***INT1***	Integrin-like protein 1	KESISSKPAKLSSASPR	7.00E-11	100%
		DLNFANYSNNTNRPR	2.00E-10	100%
***LIP4***	Secreted lipase	IFKSGWNILKNPTISK	1.00E-11	100%
***orf19*.*4984***	C1_13530wp_a; Pseudogene; has lysine motifs associated with chitin binding	SGIDTTKVYGGLANYGR	3.00E-10	94.1%
***PGA14***	Putative GPI-anchored protein; induced during cell wall regeneration	KFTTVATVFAISSLAAAKGGEK	3.00E-16	100%
***PGA27*** ^a^	Predicted GPI-anchored protein of unknown function	DGVPINFHRAPAIIMK	8.00E-12	100%
***PGA4***	GPI-anchored cell surface protein; beta-1,3-glucanosyltransferase	YFQELGINTIRVYSIDNTK	1.00E-14	100%
***SOD4***	Cu-containing superoxide dismutase	NSTNGSSGSSTSASQGSGAGR	7.00E-15	100%
***SSU81***	High osmolarity signaling protein	NTIYTDSETGTGITFR	4.00E-11	100%
***STP2***	Amino-acid-regulated transcription factor	HFNYAKPIKSAERSK	2.00E-10	100%
***XOG1***	Exo-1,3-beta-glucanase; 5 glycosyl hydrolase family member	QLSFILTSSVFILLLEFVK	2.00E-13	100%

^a^ Protein identified through MASCOT search as well from cells grown under same conditions.

**Table 5:** Proteins, extracted from cells of the *pir32* null strain grown under filamentous conditions, identified through BLAST.

Gene Name	Description	Sequence	E-value	% Match
***CDC42***	Rho-type GTPase; Cell division control protein	LSPITQEQGEKLAKELR	3.00E-11	100%
***DCW1***	Mannan endo-1,6-alpha-mannosidase	NSVSNGALFHLAARLAR	3.00E-11	100%
***DFI1*** ^a^	Cell-surface associated glycoprotein	LSINNNNNNR	2.00E-04	100%
***EXG2***	GPI-anchored cell wall protein, Glucan 1,3-beta-glucosidase 2	VGSCAEFNKSPDK	4.00E-08	100%
		NGIMPQPLDNYK	1.00E-07	100%
***KAR2***	Hsp70 family ATPase	DAGTIAGLNVLR	5.00E-06	100%
***LIP5***	Cold-activated secreted lipase	QTVSGCQHIQR	6.00E-07	100%
***GA26***	GPI-anchored adhesin-like protein of the cell wall	GGSSSGSSSGSSSGSRGGSSSGSSSSGSR	3.00E-22	100%
		NVAGALVGVVAIAAAMM	5.00E-10	100%
***PGA32***	Putative GPI-anchored adhesin-like protein	ITPIASASASSGSSTK	1.00E-09	100%
***PGA37***	Putative GPI-anchored protein	GGSSSGSSSGSSSGSRGGSSSGSSSSGSR	3.00E-22	100%
***PGA49*** ^a^	Predicted GPI-anchored protein 49 of unknown function	LDKPKFPDIFTIIR	2.00E-09	100%
***PMT5***	Protein mannosyltransferase	FSFWSKLIETHK	7.00E-08	100%
***RBT5*** ^a^	Repressed by TUP1 protein 5	AEETSKAAETTK	7.00E-06	100%
***RPL13*** ^a^	60S ribosomal protein L13	AISKNLPLLNNHFRK	5.00E-10	100%
***SAP3***	Secreted aspartyl proteinase 3	NVTGPQGEINTNVNVK	8.00E-11	100%
***SOD6***	Copper-containing superoxide dismutase	HGCINTTCFELK	2.00E-08	100%
		EGKHVNVHIDMTGLPK	1.00E-11	100%
***TPI1***	Triose-phosphate isomerase	VILCIGETLEER	9.00E-07	100%
***UCF1***	Upregulated by cAMP in filamentous growth	LELDVSCTNESAMVDVEYKSIPM	1.00E-18	100%
		FRRDLQHHIQK	5.00E-07	100%

^a^ Protein identified through MASCOT search as well from cells grown under same conditions.

**Table 6:** Proteins exclusively detected in the wild type strain under each growth condition.

Gene Name	Description
**Wild type strain grown under nonfilamentous condition**	
***RPL13***	60S ribosomal protein
***MPG1***	Mannose-1-phosphate guanyltransferase; required for glycosylation and cell wall synthesis
***QDR2***	MFS antiporter that does not act as drug transporter but performs functions that significantly affect biofilm development and needed for growth in the host
***EGD2***	Mitochondrial outer membrane component
***LIP9***	Secreted lipase, has a role in nutrition and in creating an acidic microenvironment
***CBR1***	Electron donor reductase for cytochrome b5 that supports the catalytic activity of several sterol biosynthetic enzymes
***PGA1***	GPI-anchored protein of cell wall involved in virulence
***CHT2***	Chitinase involved in the remodeling of chitin in the fungal cell wall
***RPS1***	Putative ribosomal protein 10 of the 40S subunit
***PIR32***	Cell wall protein
***IFF6***	Adhesin-like protein
IFF11*	Secreted protein required for normal cell wall structure and for virulence
FMP13*	Mitochondrial inner membrane component
PGA30*	GPI-anchored protein of cell wall with an unknown function
PGA45*	GPI-anchored protein of cell wall with an unknown function
RIM9*	Plasma membrane protein, required for alkaline pH response
**Wild type strain grown under filamentous condition**	
***LIP4***	Secreted lipase, has a role in nutrition and in creating an acidic microenvironment
***CHT2***	Chitinase involved in the remodeling of chitin in the fungal cell wall
***HYR4***	Putative GPI-anchored adhesin-like protein
***PGA53***	GPI-anchored cell surface protein of unknown function
***QDR3***	MFS antiporter that does not act as drug transporter but performs functions that significantly affect biofilm development and needed for growth in the host
***PIR32***	Cell wall protein
PRA1*	pH regulated antigen, a cell surface protein that sequesters zinc from host tissue and is enriched at hyphal tips
FMP39*	Mitochondrial inner membrane component
QCR2*	Component of the ubiquinol-cytochrome c reductase complex which is part of the mitochondrial respiratory chain
QDR2*	MFS antiporter that does not act as drug transporter but performs functions that significantly affect biofilm development and needed for growth in the host
RBR1*	Glycosylphosphatidylinositol (GPI)-anchored cell wall protein required for filamentous growth at acidic pH
SRB1*	GDP-mannose pyrophosphorylase involved in cell wall synthesis where it is required for glycosylation

The * symbol indicates proteins identified through BLAST search.

**Table 7:** Differentially detected hyphae-specific proteins in the *pir32* null strain between filamentous growth and nonfilamentous growth.

Gene Name	Description
***pir32* null strain grown under filamentous condition**	
***ALS3***	Hyphal cell wall specific adhesin and invasin
***CSA2***	Extracellular heme-binding protein involved in heme-iron acquisition, required for normal RPMI biofilm formation
***ECE1***	Extent of cell elongation, functions as a candidalysin
***FBA1***	Fructose-bisphosphate aldolase, a glycolytic enzyme that is antigenic
***RPS1***	Putative ribosomal protein 10 of the 40S subunit, also located in hyphal cell wall not yeast
***TPI1***	Triose-phosphate isomerase, antigenic, mutation affects filamentation
***UTR2***	Extracellular glycosidase functions in fungal pathogenesis, cell wall assembly and regeneration, filamentation, and adherence to host cells
***pir32* null strain grown under nonfilamentous condition**	
***BUD2***	Protein important to maintain linear growth by stabilization of the polarisome at hyphal tips and necessary for thigmotropism
***CDC11***	Septin that plays a key role in invasive growth and virulence
***DFG5***	Required for normal synthesis of the cell wall and alkaline pH-induced hypha formation
***ENO1***	Enolase, functions in glycolysis and gluconeogenesis and is a major cell-surface antigen
***HWP2***	GPI-anchored cell wall protein required for mating efficiency, biofilm formation, adhesion, filamentous growth, and oxidative stress tolerance
***INO1***	Inositol-1-phosphate synthase
***PDC11***	Pyruvate decarboxylase, antigenic
***SSA2***	HSP70 family chaperone

**Table 8:** Differentially detected hyphae-specific proteins in the wild type strain between filamentous growth and nonfilamentous growth.

Gene Name	Description
**Wild type strain grown under filamentous condition**	
***ECE1***	Extent of cell elongation, functions as a candidalysin
***PRA1***	pH regulated antigen, a cell surface protein that sequesters zinc from host tissue
**Wild type strain grown under nonfilamentous condition**	
***HYR1***	GPI-anchored hyphal cell wall protein involved in resistance to killing by neutrophils
***RBT1***	Cell wall protein with similarity to Hwp1, required for virulence

### 3.1 Proteins exclusively detected in *pir32* null strain as identified through MASCOT

#### 3.1.1 Under non-filamentous growth

Table 2 shows the proteins that were extracted under non-filamentous growth conditions and that were identified only in the mutant. Six proteins were identified. Note the presence of Dfg5 which is an N-linked cell wall mannoprotein with roles in cell wall biogenesis and organization, Dse1 a protein of the cell wall responsible for its integrity and rigidity, virulence, and cell adhesion, Nce102 which functions in actin cytoskeleton organization, and Sap2 which is an aspartyl proteinase functioning in adhesion to the host and virulence.

#### 3.1.2 Under filamentous growth

Table 3 shows the proteins extracted under filamentous growth conditions and that were identified only in the mutant. A total of 12 proteins were identified. Note the presence of Als3 an agglutinin-like protein functioning in epithelial adhesion and endothelial invasion as well as biofilm formation, Csa2 a surface antigen protein required for biofilm formation, Dfi1 a cell-surface associated glycoprotein needed for cell wall maintenance and invasive filamentation, Ssr1 a beta-glucan having a role in cell wall structure, and multiple cell wall proteins that are members of the PGA (putative GPI-anchor) family.

### 3.2 Proteins exclusively detected in *pir32* null strain as identified through BLAST

#### 3.2.1 Under non-filamentous growth

A total of 11 proteins, listed in Table 4, were identified only in the mutant grown under non-filamentous conditions through BLAST searching the peptide sequences generated by MASCOT against *C*. *albicans* database. Note the presence of Int1 that has a role in virulence, C1_13530wp_a that has a role in chitin binding, Sod4 that affects the response to oxidative stress, Ssu81 that has roles in invasion, response to oxidative stress, and cell wall organization, Xog1 that affects cell wall integrity and cell adhesion, and several PGA family proteins with role in cell wall structure.

#### 3.2.2 Under filamentous growth

In Table 5, 16 proteins are listed and these were identified only in the mutant under filamentous growth conditions after BLAST searching the MASCOT peptides across the *C*. *albicans* database. The most prominent of these proteins are Cdc42 and Sap3 involved in virulence, Dcw1 required for the synthesis of the cell wall, Exg2 involved in the metabolism of beta glucan and cell wall organization, Lip5 affecting biofilm formation, Pga26 involved in cell wall integrity and required for virulence, and Sod6 affecting the response to oxidative stress.

### 3.3 Proteins exclusively detected in the wild type strain

A total of 16 proteins were exclusively detected in the wild type strain under nonfilamentous growth and a total of 12 under filamentous growth as presented in Table 6. Under nonfilamentous growth, 11 proteins were identified by MASCOT and the remaining 5 were identified by BLAST. Under filamentous growth, half the proteins were identified by MASCOT and the other half through BLAST.

### 3.4 *pir32* null hyphae-specific cell wall proteins

Upon comparing the cell wall proteome profiles of the *pir32* null strain between filament and nonfilament inducing growth conditions, a total of 8 proteins were found under nonfilamentous growth as seen in Table 7.

### 3.5 Wild type hyphae-specific cell wall proteins

When comparing the cell wall proteome profiles of the wild type strain between filamentous and nonfilamentous growth, 2 proteins were detected under the latter state as seen in Table 8.

## 4 Discussion

This study aimed at further characterizing the *C*. *albicans* cell wall mannoprotein Pir32. A previous study found that Pir32 is involved in virulence and cell wall integrity of *C*. *albicans* [13]. Furthermore the *pir32* null strain was previously found to have higher cell wall chitin deposition and to be more virulent, more resistant to disrupting agents and oxidative stress, and hyper-filamentous when compared to the wild type strain. Cell wall protein extraction followed by MALDI mass spectrometry coupled with database searches allowed for identification of proteins that could explain these phenotypes as they were solely detected in the mutant strain. It is crucial to note that the inability to detect a protein in a certain strain does not strictly entail its absence from the sample. However, it implies that it is found at a much higher concentration in one strain versus the other, resulting in the observed phenotypes.

The majority of the extracted proteins are cell wall localized proteins, however some proteins identified through BLAST were cytosolic. The presence of such proteins might be due to the incomplete separation of the cell wall from the other cellular components and the subsequent contamination. However, proteins like Kar2 and Rpl13 have been previously termed “atypical” *C*. *albicans* cell wall proteins since, although they were previously found to be members of cytosolic pathways and lack the structural properties of genuine cell wall proteins, they are being identified within cell wall extracted protein fractions. Thus, it was proposed that they are non-covalently bound to and retained in the cell wall [9, 20].

A total of 11 proteins detected only in the mutant strain are members of the PGA family that contains almost 65 proteins, most of which are uncharacterized yet [21]. Pga27, Pga37, and Pga49 have unknown functions. Pga32 is an adhesin that plays a role in cell adhesion while Pga14 and Pga17 were found to be involved in cell wall regeneration and virulence. Pga31 was previously found to be involved in chitin deposition which in turn affects cell wall integrity and resistance to perturbing agents [22]. The presence of this protein in the mutant could explain the twofold increase in chitin content in the cell wall. Pga4 is a glucanosyl transferase that is involved in cell wall remodeling and integrity due to its role in β-1,3-glucan chains’ assembly and elongation [23]. Pga26 is a glycoprotein that maintains cell wall integrity and plays crucial roles in the resistance of *C*. *albicans* to stress and antifungal drugs [24]. Also, Sod4 and Sod6, known as Pga2 and Pga9 respectively, are superoxide dismutases that aid in evading the immune surveillance of the host by detoxifying the reactive oxygen species produced by this host [25]. This implies that the identified PGA proteins are responsible for the increase in chitin deposition, cell wall integrity, resistance to oxidative stress and disrupting agents, virulence, and subsequent pathogenesis of the mutant.

Many of the identified proteins play a crucial role in cell wall biosynthesis and organization as well. It has been previously shown that occasionally a cell compensates for a cell wall protein deletion by increasing the thickness and rigidity of the cell wall [22]. Ssr1 is a GPI-anchored protein that contributes to the assembly of cell wall components and its overall organization [26]. Xog1 is a member of the 5 glycosyl hydrolase family that plays an important role in the metabolism of beta-glucan, delivery of glucan to the extracellular matrix, and adhesion to the host [27]. Dfg5 and Dcw1 are mannosidases required for cell wall synthesis [28]. Cell wall organization and chitin content are important factors for maintaining cell wall integrity required for resisting any stress. Accordingly, the detection of these proteins in the mutant strain clarify its higher resistance to SDS and osmotic stress than the wild type.

Another distinctive phenotype of the mutant was its hyperfilamentation. Several proteins found in the mutant are related to invasive filamentation and morphogenesis such as Cdc42, Dfi1, Ssu81, Int1, and Ucf1. The most notable of these is Ssu81, an osmosensor at the plasma membrane [29]. This protein has a major role in pathogenesis and is a known mediator of oxidative stress resistance that acts by affecting the polymer distribution and molecular weight of cell wall mannan, increasing invasive filamentation, and activating the dimorphic transition [30]. Int1 is required for the axial budding of yeast cells and for allocating the septation sites in hyphal cells. This protein induces hyphal growth and promotes morphogenesis [31]. Cdc42 is required for budding and hyphal growth [32]. Dfi1 is a glycoprotein that binds calmodulin upon matrix sensing which initiates a signaling cascade that in turn promotes the formation of invasive filaments [33]. Collectively, the detection of these proteins exclusively in the *pir32* null strain explain its hyperfilamentous phenotype.

Switching from yeast to hyphae is required for fungal fitness and adaptation to its environment. It has been proposed that upon nutrient deprivation, *C*. *albicans* cells start catabolizing amino acids as a source for carbon, thus excreting ammonia which raises the pH in its environment and activating morphogenesis [34]. In the mutant, the 4 identified lipases (Lip4, 5, 7 and 9) and 2 aspartyl proteases (Sap2 and 3) along with Stp2 contribute to this process as they are enzymes that degrade the host cell membrane and proteins [35–37]. The ability of *C*. *albicans* to form invasive filaments and use nutrients from the host is a major virulence factor.

Iron utilization is another virulence factor that allows invading microorganisms to survive the host defense mechanisms, mainly iron withholding, and proliferate in its tissues. *C*. *albicans* became a successful pathogen by scavenging iron molecules from the host [38]. Rbt5, a GPI-anchored protein, is a heme receptor and a major protein in the heme-iron acquisition pathway [39]. Csa2 is a secreted protein that also plays a role in heme-iron utilization. Both proteins were detected in the mutant and not identified in the wild type; thus explaining the increased virulence and pathogenesis of the mutant strain [40]. In our study, we also detected Als3 in the mutant as well. This adhesin participates in ferritin-iron binding along with adhesion, aggregation and subsequently pathogenesis [41].

Exg2, Tpi1, and Dse1 are all essential proteins that were only detected in the mutant strain in our study although they should have been common between both strains. This however does not imply their total absence from the wild type samples; they might be present at very low concentrations. Accordingly, we hypothesize that these proteins have been overexpressed as a compensation for the deletion. These proteins participate in cell wall regeneration and rigidity, filamentation, and virulence thus reflecting the observed phenotypes of the mutant [27, 42, 43].

Most of the proteins exclusively detected in the wild type strain are either artifacts or atypical proteins since they are known to be cytosolic proteins, ribosomal subunits, or mitochondrial components as seen in Table 6. The remaining are cell wall proteins with functions related to cell wall synthesis, chitin remodeling, and virulence. This is expected since the wild type strain is virulent and many proteins involved in pathogenicity are incorporated in its cell wall.

Upon comparing proteins from the *pir32* null strain but under filamentous versus nonfilamentous conditions, many hyphae-specific proteins were detected under nonfilamentous growth which highlight its hyperfilamentous phenotype. This strain was able to grow filaments under nonfilament-inducing conditions and this is explained by the incorporation of several hyphae-specific proteins in its cell wall as listed in Table 7. Interestingly, we found 2 hyphae-specific cell wall proteins, Hyr1 and Rbt1, in the wild type strain under nonfilament inducing conditions.

The detection of all of the above mentioned proteins in the mutant can explain the enhanced levels of filamentation, chitin content, virulence, and resistance to stress and perturbing agents previously observed. It is worth noting that in a previous analysis of *a pir 32* null strain, we found the mutant to have upregulated chitin deposition two fold to overcompensate the weakening of the cell surface upon deletion of *pir 32* [13]. The large number and nature of the proteins detected exclusively in our mutant also tends to suggest that in addition to increased chitin deposition our strain also upregulated genes involved in virulence and pathogenicity to compensate for the deletion. All in all our study indicates that Pir 32 plays an important role in the cell surface architecture of *C*. *albicans*.
